# Regulating carbon and water balance as a strategy to cope with warming and drought climate in *Cunninghamia lanceolata* in southern China

**DOI:** 10.3389/fpls.2022.1048930

**Published:** 2022-11-17

**Authors:** Xuan Fang, Tian Lin, Biyao Zhang, Yongru Lai, Xupeng Chen, Yixin Xiao, Yiqing Xie, Jinmao Zhu, Yusheng Yang, Jian Wang

**Affiliations:** ^1^ Fujian Provincial Key Laboratory for Plant Eco-physiology, Fujian Normal University, Fuzhou, China; ^2^ School of Life Sciences, Fujian Normal University, Fuzhou, China; ^3^ Fujian Sanming Forest Ecosystem National Observation and Research Station, Sanming, China; ^4^ School of Ecological Environment and Urban Construction, Fujian University of Technology, Fuzhou, China; ^5^ Institute of Economic Forestry, Fujian Academy of Forestry, Fuzhou, China; ^6^ State Key Laboratory for Subtropical Mountain Ecology of the Ministry of Science and Technology and Fujian Province, Fujian Normal University, Fuzhou, China; ^7^ School of Geographical Sciences, Fujian Normal University, Fuzhou, China

**Keywords:** warming, drought, carbon and water balance, defoliation, Chinese fir

## Abstract

Human activities have increased the possibility of simultaneous warming and drought, which will lead to different carbon (C) allocation and water use strategies in plants. However, there is no conclusive information from previous studies. To explore C and water balance strategies of plants in response to warming and drought, we designed a 4-year experiment that included control (CT), warming (W, with a 5°C increase in temperature), drought (D, with a 50% decrease in precipitation), and warming and drought conditions (WD) to investigate the non-structural carbohydrate (NSC), C and nitrogen (N) stoichiometry, and intrinsic water use efficiency (iWUE) of leaves, roots, and litter of *Cunninghamia lanceolata*, a major tree species in southern China. We found that W significantly increased NSC and starch in the leaves, and increased NSC and soluble sugar is one of the components of NSC in the roots. D significantly increased leaves’ NSC and starch, and increased litter soluble sugar. The NSC of the WD did not change significantly, but the soluble sugar was significantly reduced. The iWUE of leaves increased under D, and surprisingly, W and D significantly increased the iWUE of litter. The iWUE was positively correlated with NSC and soluble sugar. In addition, D significantly increased N at the roots and litter, resulting in a significant decrease in the C/N ratio. The principal component analysis showed that NSC, iWUE, N, and C/N ratio can be used as identifying indicators for *C. lanceolata* in both warming and drought periods. This study stated that under warming or drought, *C. lanceolata* would decline in growth to maintain high NSC levels and reduce water loss. Leaves would store starch to improve the resiliency of the aboveground parts, and the roots would increase soluble sugar and N accumulation to conserve water and to help C sequestration in the underground part. At the same time, defoliation was potentially beneficial for maintaining C and water balance. However, when combined with warming and drought, *C. lanceolata* growth will be limited by C, resulting in decreased NSC. This study provides a new insight into the coping strategies of plants in adapting to warming and drought environments.

## Introduction

The Intergovernmental Panel on Climate Change (IPCC) has shown that human actions contribute to a gradual increase in global average surface temperature due to elevated emissions of greenhouse gases such as carbon dioxide (CO_2_), methane (CH_4_), and nitrous oxide (N_2_O) (IPCC, 2021). Human actions increase atmospheric evaporation and increase the possibility of droughts (IPCC, 2021). In current and future climate change, warming and drought may occur simultaneously and are expected to affect plant physiology and ecosystem function (Auyeung et al., 2013; Zhang et al., 2021). These effects include combined effects on plant foliar chemistry (Orians et al., 2019) as well as changes in carbon (C) balance and water balance (Mystakidis et al., 2016).

Carbon (C) plays a central role when plants are grown in negative environments because it is one of the most abundant and versatile elements involved in plant metabolism (Hartmann et al., 2020). Non-structural carbohydrates (NSC) can reflect the C balance between photosynthetic C assimilation and metabolic C demand in woody plants (Kannenberg and Phillips, 2019) and include soluble sugar (e.g., glucose and sucrose) and starch. Plants through newly absorbed C and stored NSC provide growth and other physiological functions such as respiration, osmoregulation, and defense mechanisms (Hartmann and Trumbore, 2016; Gersony et al., 2020; Trowbridge et al., 2021; Liu et al., 2022). At the same time, NSC is an important reserve substance that can be used in stressful environments (Yang et al., 2018), such as drought, warming, and excessive CO_2_ (Xu et al., 2013; Fernández de Simón et al., 2022). The NSC can reflect the C equilibrium status of the plants when grown in a negative environment. Therefore, identifying the changes of NSC storage under adverse conditions not only is key for understanding C dynamics in trees in the context of global climate change, but also has broader implications for ecosystem function and the prediction of forest responses to global change (Katarína et al., 2019).

NSC is of particular interest as they are the dominant currency of C allocation and they are also a critical indicator of C limitation of trees (Hartmann et al., 2020). Currently, drought is one of the most crucial abiotic stresses affecting plants and can affect a range of physiological processes in trees, which, in turn, affects their growth and productivity (Seleiman et al., 2021). At present, various studies have reported the impact of drought on NSC, but there are conflicting results regarding NSC dynamics during drought (Mitchell et al., 2013; Lin et al., 2018). Therefore, further investigations are needed to better understand and predict adaptation strategies of plant C balance to global climate scenarios. At the same time, warming may have deleterious consequences on subtropical and tropical forests because numerous tree species occur near the thermal optimum due to climate change (Li et al., 2016). It has been shown that at high temperatures, a decrease in photosynthesis due to higher respiration, stomatal closure, may lead to a rapid depletion of the C storage pool (Zhao et al., 2013). Similar to drought, the effects of warming on plant NSC are not obviously established (Shi et al., 2015; Danielle et al., 2016; Tang et al., 2016; Zheng et al., 2018). Most studies thus far have focused only on the response of NSC to individual plant tissues to environmental stresses (Michael et al., 2014; Hartmann and Trumbore, 2016). This can lead to inconsistent results, as different tissues may have different C allocation strategies. Changes in NSC concentration between different plant organs can reflect the allocation strategy of NSC under negative C balance (He et al., 2020; D'Andrea et al., 2021; Guo et al., 2021). At present, the mechanism of the effect of drought on NSC is essentially understood, but adaptation strategies of C balance under drought and how warming affects the concentration and distribution of NSC in plants remain unclear. In addition, studies have shown that carbon in forest ecosystems may be vulnerable to the combined effects of drought and warming (Bonan, 2008). In particular, subtropical forests may be affected by additional warming and frequent droughts (Ma et al., 2017; Zhang et al., 2019). Therefore, quantitative studies of the contribution of NSC to the C balance under warming and drought conditions are essential for understanding the survival and growth of subtropical plants.

Intrinsic water use efficiency (iWUE) is defined as the ratio of the photosynthetic uptake of CO_2_ to the simultaneous transpiration loss of water vapor, both through the stomata (Farquhar et al., 1982a). Previous studies have shown that plants can improve their tolerance by increasing the iWUE values to help them grow under drought stress as water stress increases (Churakova et al., 2020; Yang et al., 2021). At the same time, high temperature may reduce stomatal conductivity and the transpiration rate of trees in leaves and increase iWUE (Granda et al., 2014; Hararuk et al., 2019), but the increase in iWUE did not offset the negative impact of warming on tree growth (Ren et al., 2022), which drove growth declines of about 50% (Heilman et al., 2021). In the course of plant life, water and carbon are so closely connected that it is difficult to separate them. Further research is needed to understand the relationship between iWUE and C, and strategies for C and water adaptation in a warming and drought environment. This knowledge gap has limited the understanding of the forest C and water cycle and the exploration of its mechanisms.

Under drought stress, trees mainly prevent water loss by reducing stomatal conductance and assimilation rate, while controlling C uptake by plants (Farquhar and Sharkey, 1982b; Adams et al., 2009). Meanwhile, the trees’ stored NSC has a potential role in increasing tolerance and maintaining survival (Francisco et al., 2018; Lin et al., 2018). iWUE serves as an indicator of stomatal responses to environmental variability and is indicative of the trade-off between C uptake and water loss (Kannenberg et al., 2021). If trees have water loss to promote tree growth, they would not be able to cope with increased drought. Hence, competition forces the trees to have a trade-off relationship between carbon uptake and water loss (Zhang et al., 2022). Tree-scale iWUE is affected by the allocation of C to different organs, and the formation, storage, and utilization of NSC to regulate plant metabolism (Hartmann and Trumbore, 2016). Therefore, the NSC and iWUE may cooperate to cope with environmental stress. Warming and drought affect carbon and water fluxes and their coupling relationships in ecosystems. Understanding how carbon and water respond to warming and drought conditions can help us predict future forest adaptation strategies under global climate change conditions.

To better explore the C and water adaptive strategies of woody plants in future warming and drought environments, the study focused on Chinese fir [*Cunninghamia lanceolata* (Lamb.) Hook.], one of the most valuable timber species in southern China, which is a typical subtropical coniferous tree species. This study is also significant for the carbon budget of terrestrial ecosystems (Yu et al., 2016). A 4-year experiment studied the effects of artificial soil warming (5°C) and isolation of 50% rainfall on tree C and water balance by investigating the NSC, iWUE, and C and N stoichiometry in multiple tissues (leaves, roots, and litter). This study aimed to answer the following questions: (1) How do the effects of warming and drought stress affect NSC and their composition in plants? (2) How do plants regulate their own C and water balance to adapt to warming and drought stress?

## Materials and methods

### Study sites

**Figure 1:**
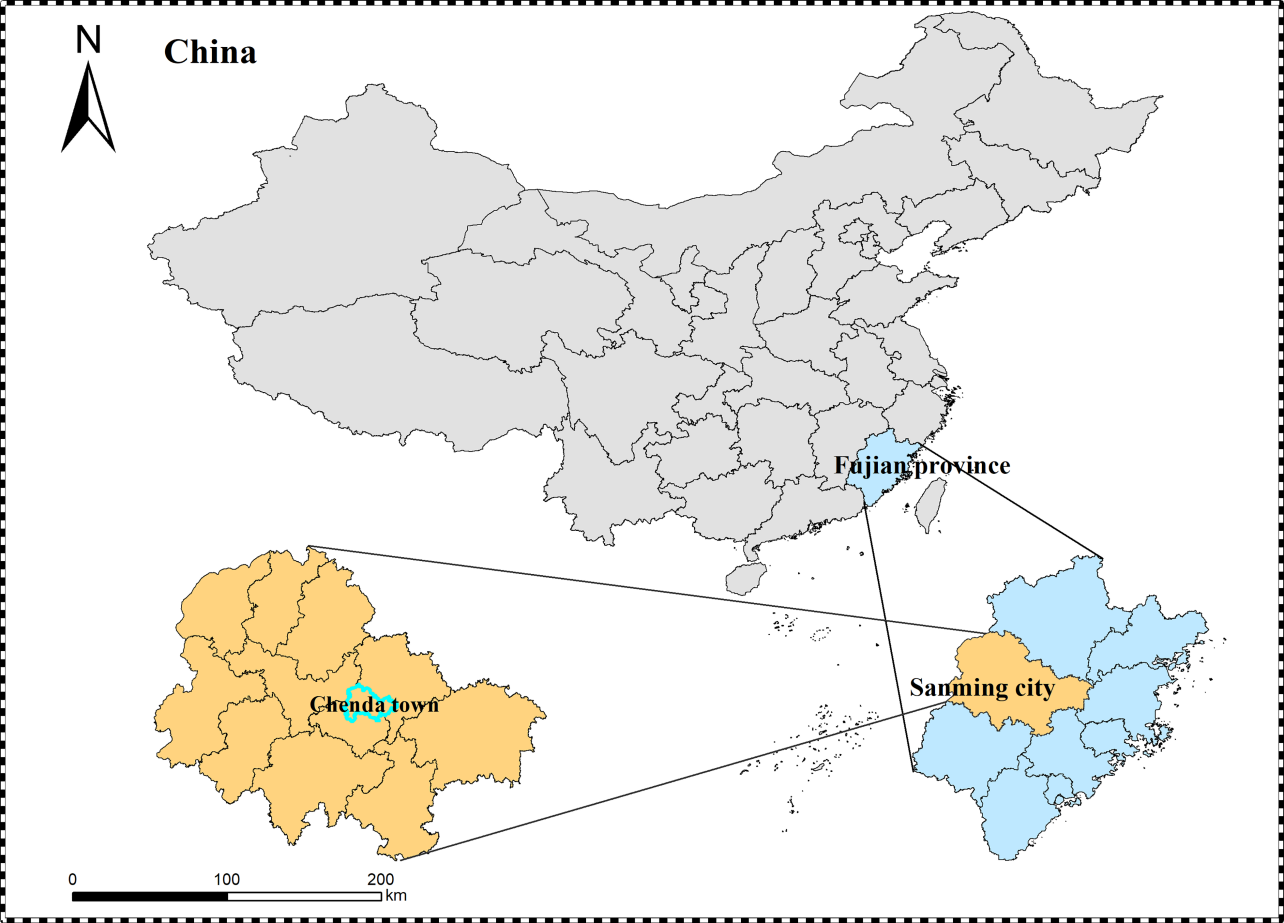
Map of the study area.

The study was carried out in the Forest Ecosystem and Global Change National Observation and Research Station of Fujian Normal University in Chenda Town (26°19′ N, 117°36′E), Sanming City, Fujian Province, China (**Figure 1**). The climate was characterized as a subtropical monsoon. The study site had a mean annual rainfall of 1,670 mm, which mainly fell from March to August, and the mean annual temperature was 19.1°C, besides a relative humidity of 81%. The soil has been classified as clay, gibbsite mixed, thermal, and Typic Hapludult. The elevation is 300 m above sea level.

### Experimental design

The experiment was a randomized complete block factorial design in twenty 2 m × 2 m mesocosms, including four treatments with five mesocosms per treatment. Factor levels were combined in four treatments: non-warming and non-isolated precipitation (CT), elevated temperature (5°C above the ambient soil temperature) and non-isolated precipitation (W), ambient temperature and isolated precipitation 50% (D), and elevated temperature and isolated precipitation 50% (WD).

The mesocosm and the surrounding soil were separated by four PVC boards (200 cm × 70 cm deep) to prevent mutual interference. In October 2013, heating cables with a depth of 10 cm and a spacing of 20 cm (the same cables were laid in all the communities) were laid in parallel. Soil temperature was measured using temperature sensors (T109; Campbell Scientific Inc., Logan, UT, USA) buried continuously between the heating cables in each mesocosm. The soil temperature significantly increased (5°C) in the warmed mesocosms at 10 cm depth by using warming cables, and the effects of the cables over the soil surface were equal to those of the control (Zhang et al., 2019).

In November 2013, 80 C*. lanceolata* seedlings with an average height of (25.7 ± 2.52) cm and an average trunk base diameter of (3.35 ± 0.48) cm were selected and randomly transplanted into the test mesocosms: in each mesocosm, four trees were planted. The drought experiment was carried out by excluding 50% of the precipitation with a rain shelter. The specific physical and chemical properties of soil were as follows: In the four treatments, the average soil moisture varied considerably throughout and, relative to CT, it decreased by 14.3%, 16.0%, and 28.8% in D, W, and WD, respectively. The soil temperature of the W and WD treatments increased significantly compared with the CT treatment. Soil pH and total C and N were not significantly different, whereas available N increased significantly by 21.2% in WD (Zhang et al., 2019).

### Experimental material

Samples were collected in April 2018. Leaves were sampled: trees in the south-facing part of the mesocosm were selected, and 40 fully expanded leaves of the same height were randomly collected from the branches, while litter was collected from the ground and placed in marked envelopes. The roots were sampled: an inner growth ring (diameter 20 cm, depth 20 cm) is placed in the center of each mesocosm. The inner growth ring is smashed into the soil, the soil in the ring is dug out and quickly brought back to the laboratory, all the roots are picked out and washed with water, and the living roots are picked out and placed in a marked envelope. All samples were oven-dried at 65°C for 72 h. Dried samples were ground to a powdered form using a mortar and pestle and passed through a 0.149-mm sieve before measuring C, N, δ^13^C, and NSC.

### Elemental analysis

Samples’ C and N concentrations were measured using a CN auto analyzer (Vario Max CN, Elementar, Langenselbold, Germany). Stable isotopic analysis for C was performed using an isotope ratio mass spectrometer (Finnigan MAT-253; Thermo Electron Crop., San Joss, CA, USA) coupled to an automatic online elemental analyzer (Flash EA1112; Electron Crop).

### Stable isotope analysis

To evaluate short-term iWUE, we performed δ^13^C analyses. The δ notation was used to express stable isotopic abundances per mille (‰) relative to international standards:


(1)          δ13Csample(‰)= Rsample−RstandardRstandard x 1000   

where R_sample_ is the value of sample ^13^C/^12^C, and R_standard_ is the value of the standard material ^13^C/^12^C.

Beginning with raw δ^13^C measurements, Eqs. (2) and (3) were used to calculate iWUE.


(2) $$\text{ △ }{\;}^{13}\text{C= }\frac{{\text{δ}}^{13}{\text{C}}_{\text{air}}-{\text{δ}}^{13}{\text{C}}_{\text{sample}}}{{1+\delta }^{13}{\text{C}}_{\text{sample}}}\;$$

where δ^13^C_air_ and δ^13^C_sample_ denote the δ^13^C values of atmospheric CO_2_ and the sample, respectively. The average δ^13^C_air_ was obtained from Antarctic ice core data (Mccarroll and Loader, 2004); Δ is related to the intercellular CO_2_ concentration (C_i_) and the ambient CO_2_ concentration (C_a_), as described by Farquhar et al. (1989) as follows:


(3) $$\;\Delta^{13}\text{C= a }+\;\left(\text{b }-\text{a}\right)\frac{{\text{C}}_{\text{i}}}{{\text{C}}_{\text{a}}}\;$$

where a is the discrimination against ^13^CO_2_ during CO_2_ diffusion through the stomata (a = 4.4‰) and b is the discrimination associated with carboxylation (b = 27‰). The iWUE was calculated using Eq. (4):


(4) $$\text{ iWUE=}\frac{\text{A}}{{\text{g}}_{\text{s}}}=\frac{{\text{C}}_{\text{a}}\;-{\text{ C}}_{\text{i}}}{1.6}=\frac{{\text{C}}_{\text{a}}\left(\text{b }-\;\Delta^{13}\text{C}\right)}{1.6\left(\text{b }-\text{ a}\right)}\;$$

where 1.6 is the ratio of the gaseous diffusivity of CO_2_ to the water vapor and the Ca value is from Zhang et al. (2019).

### NSC analysis

The concentration of NSC is the sum of soluble sugar and starch. The NSC concentration was determined using a modified phenol–sulfuric acid method (Buysse and Merckx, 1993; Zhao et al., 2021).

Preparation of sucrose standard solution: sucrose was baked to a constant weight at 80°C, weighed to the nearest 100 mg using a digital balance (accurate to 0.0001 g), dissolved in distilled water, and poured into a 100-ml volumetric flask. Next, a standard solution of sucrose was prepared at concentrations of 20, 40, 60, 80, and 100 g/L, and the absorbance was measured at 490 nm with an ultraviolet spectrophotometer to construct a standard curve.

Extraction of soluble sugar: Dried sample powder (60 mg) was extracted with 10 ml of 80% ethanol for 24 h and centrifuged at 4,000 rpm for 10 min, and the supernatant was poured into a 20-ml volumetric flask. Next, 5 ml of 80% ethanol was added to the residue and centrifuged for 5 min before the supernatant was transferred to a volumetric flask. The solution was diluted to 20 ml and used to determine the concentration of soluble sugar.

Extraction of starch: The residue was baked after the aforementioned extraction at 100°C for 3 h, added with 10 ml of distilled water and 3 ml of 3% HCl, placed in a boiling water bath for hydrolysis for 3 h, then filtered and diluted to 20 ml, which was used for starch content determination.

Determination of soluble sugar and starch concentration: In this step, 1 ml of the sample solution and 1 ml of 28% phenol solution (dissolved in 80% ethanol) were added to a centrifuge tube followed by immediate addition of 5 ml of concentrated sulfuric acid; next, the tube was shaken for 1 min and allowed to react or 15 min. Absorbance was measured at 490 nm with an ultraviolet-visible spectrophotometer, and concentrations of soluble sugar and starch were calculated based on the standard curve for sucrose.

### Statistical analyses

A two-way analysis of variance was used to probe the effects of warming, water stress, and their combination. Differences in mean NSC concentrations and compositions, iWUE, C and N concentrations, and ratios between different tissues were analyzed using a one-way analysis of variance. Individual treatment means were compared using the LSD test to identify whether they were significantly different at *p*< 0.05. Single linear regression models were used to compare the relationships between NSC, soluble sugar, and starch concentrations for iWUE. A principal component analysis (PCA) was performed on the data in order to examine the NSC concentrations in the three tissues and their composition with respect to C, N, and C/N ratios, as well as the contribution of iWUE to plant adaptation. All statistical analyses were carried out using the SPSS 20.0 statistical software. The figures were drawn using Origin 9.0 and Canoco 5.0 software.

## Results

### NSC concentrations and composition in different treatments

**Figure 2:**
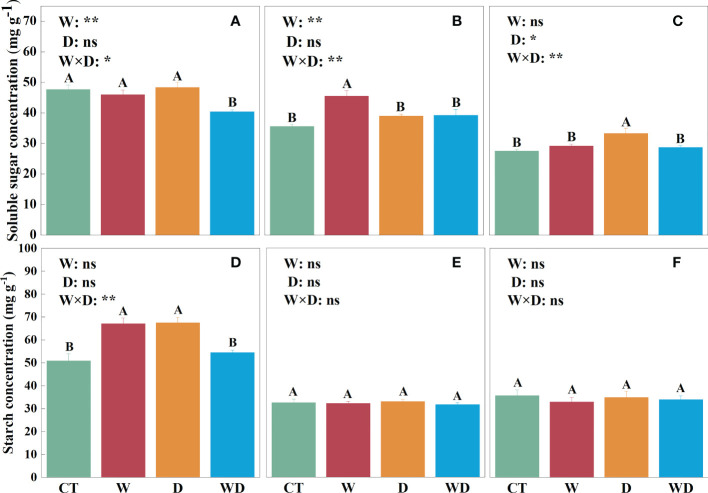
Effects of warming and drought on soluble sugar concentrations in the leaves **(A)**, roots **(B)**, and litter **(C)**, and starch concentrations in the leaves **(D)**, roots **(E)**, and litter **(F)** of *C lanceolata*. The bars with different letters were significantly different from each other (*p*< 0.05). Treatments: control (CT), warming (W), drought (D), and warming plus drought (WD). Values were mean ± SE (*n* = 5); treatment in the combination was expressed as W, warming effect; D, drought effect; and W×D, interactive effect of warming and drought; *, significant effect at *p*< 0.05; **, highly significant effect at *p*< 0.01; ns, no significant effect at *p*> 0.05.

**Figure 3:**
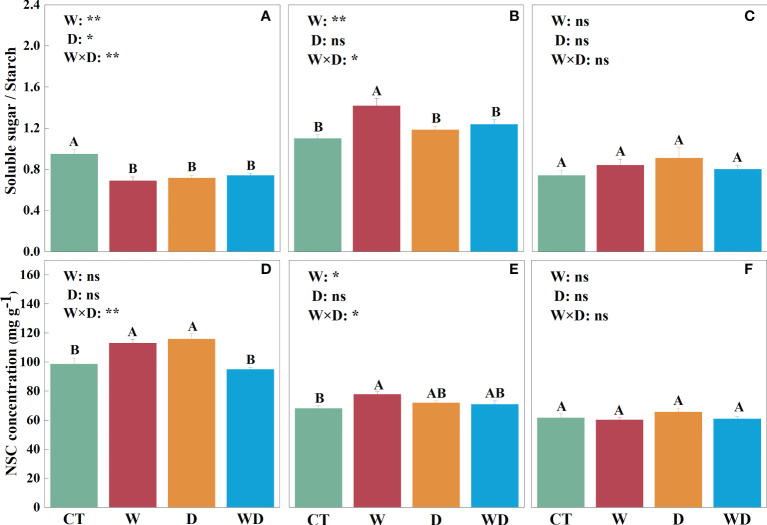
Effects of warming and drought on the ratios of soluble sugar to starch in the leaves **(A)**, roots **(B)**, and litter **(C)** and NSC concentrations in the leaves **(D)**, roots **(E)**, and litter **(F)** of *C lanceolata*. The bars with different letters were significantly different from each other (*p*< 0.05). Treatments: control (CT), warming (W), drought (D), and warming plus drought (WD). Values were mean ± SE (*n* = 5); treatment in the combination was expressed as W, warming effect; D, drought effect; and W×D, interactive effect of warming and drought; *, significant effect at *p<* 0.05; **, highly significant effect at *p*< 0.01; ns, no significant effect at *p*> 0.05.

In the leaves, WD caused a highly significant decrease in soluble sugar concentration of 15.3% compared to CT (*p*< 0.01), with no significant effect of W or D alone (**Figure 2A**). W and D alone significantly increased starch concentration by 31.9% and 32.6% (*p*< 0.01; **Figure 2D**), respectively, and the ratio of soluble sugar to starch decreased highly significantly in all three treatments (*p*< 0.01; **Figure 3A**). NSC showed the same trend as starch, with a highly significant increase of 14.8% and 17.6% in W and D alone, respectively (*p*< 0.01; **Figure 3D**). A two-way ANOVA for W and D found that all four variables changed significantly in response to the interaction of temperature increase and drought (*p*< 0.05).

In the roots, soluble sugar solutions were highly significantly increased by 27.7% in W compared to CT (*p*< 0.01; **Figure 2B**). Neither temperature increase nor water stress had a significant effect on the starch solution (**Figure 2E**). However, the ratios in roots were visibly greater than those in leaves and litter, and the ratios were greater than 1 only in roots (**Figures 3A** **–C**). The trend of NSC was similar to that of soluble sugar with a highly significant increase of 13.9% in W (*p*< 0.01; **Figure 3E**). The results of the two-way analysis indicated that all indicators except starch were significantly affected by the interplay of warming and drought.

In the litter, only soluble sugar showed a highly significant increase of 20.1% under the D treatment (*p*< 0.01; **Figure 2C**), and the interaction between W and D had a highly significant effect on them (*p*< 0.01). ​However, neither W nor D has a significant effect on starch and NSC (**Figures 2F**, **3F**).

### iWUE in different treatments

**Figure 4:**
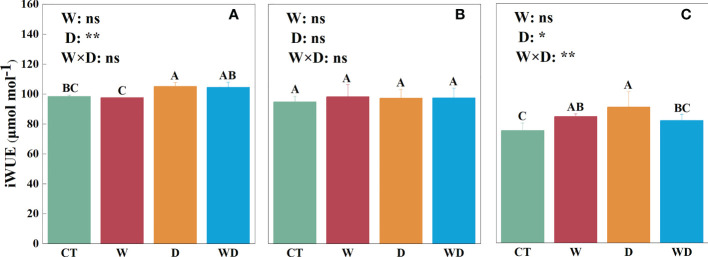
Effects of warming and drought on iWUE in the leaves **(A)**, roots **(B)**, and litter **(C)** of *C lanceolata*. The bars with different letters were significantly different from each other (*p*< 0.05). Treatments: control (CT), warming (W), drought (D), and warming plus drought (WD). Values were mean ± SE (*n* = 5); treatment in the combination was expressed as W, warming effect; D, drought effect; and W×D, interactive effect of warming and drought; *, significant effect at *p*< 0.05; **, highly significant effect at *p*< 0.01; ns, no significant effect at *p*> 0.05.

In the leaves, the D treatment significantly increased iWUE by 6.8% compared with CT (*p*< 0.05; **Figure 4A**). In the roots, iWUE showed no significant differences between either the combined effects or the individual effect (**Figure 4B**). In the litter, compared with the CT treatment, the W and D treatment significantly increased iWUE by 12.4% and 20.8%, respectively (*p*< 0.05; **Figure 4C**). The combined effects of the W and D treatments were highly significant for iWUE (*p*< 0.01; **Figure 4C**).

### Relationship between NSC, soluble sugar, starch and iWUE

**Figure 5:**
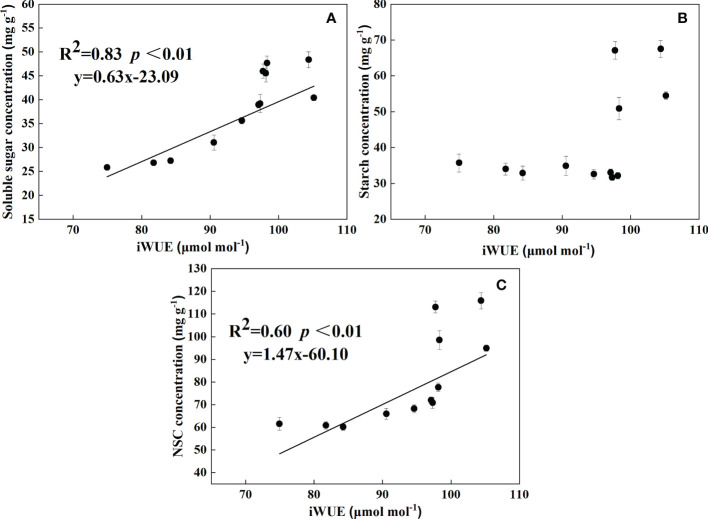
Relationships between soluble sugar **(A)**, starch **(B)**, and NSC **(C)** concentrations and iWUE in leaves, roots, and litter.

According to **Figure 5**, the concentration of soluble sugar and NSC could be used with iWUE to establish a regression model in three parts (leaf, root, and litter). The concentration of soluble sugar and NSC increased with iWUE. It showed that iWUE was significantly and positively correlated with soluble sugar and NSC, respectively. In addition, the concentrations of soluble sugar, NSC, and iWUE reached a relatively elevated value of 0.92 and 0.78, respectively. Thus, the correlation between soluble sugar and iWUE, NSC, and iWUE was strong.

### C and N stoichiometric characteristics in different treatments

In the leaves, compared with the CT treatment, the C and N concentrations and the C/N ratio were not significantly different in W, D, and their combined effects (**Table 1**). In the roots, the C concentrations were significantly different under the interaction of the W and D treatments (*p*< 0.05; **Table 1**). The N concentrations were significantly increased by 29.3% in the D treatment than in the CT treatment, which led to a significant decline of 21.4% in the C/N ratio (*p*< 0.01; **Tables 1** and **2**). In the litter, the same trend as the roots was observed: N concentrations increased by a highly significant 30.6% at the D treatment, resulting in a highly significant decrease of 21.4% in C/N ratios (*p*< 0.01; **Tables 1** and **2**).

**Table 1:** Repeated measures analysis of variance (ANOVA) of C and N concentrations and C/N ratios in leaves, roots, and litter of *C. lanceolata*.

	Source of Variations	Leaf		Root		Litter	
		*F*	*p*	*F*	*p*	*F*	*p*
C	W	0.0004	0.984	0.118	0.737	0.029	0.868
	D	0.242	0.630	1.019	0.330	1.804	0.199
	W×D	4.434	0.052	5.205	**0.039**	0.018	0.895
N	W	1.873	0.191	4.520	0.052	0.177	0.680
	D	0.033	0.858	16.778	**0.001**	15.993	**0.001**
	W×D	0.371	0.552	1.020	0.330	0.021	0.887
C/N	W	1.428	0.251	4.371	0.055	0.159	0.696
	D	0.004	0.951	23.144	**<0.01**	15.340	**0.001**
	W×D	0.432	0.521	0.619	0.444	0.025	0.876

Treatments: drought (D), warming (W), and interactive effect of drought and warming (W×D); signiﬁcant effect at p< 0.05; highly signiﬁcant effect at p< 0.01. Bold values indicate significant differences in results (P<0.05).

**Table 2:** C and N concentrations and C/N ratios in leaves, roots, and litter of *C. lanceolata*.

		Treatment (mg g^-1^)			
		CT	W	D	WD
Leaf	C	422.73 ± 3.45 A	415.15 ± 4.65 A	416.87 ± 3.28 A	424.59 ± 2.56 A
	N	4.87 ± 0.29 A	5.79 ± 0.61 A	5.07 ± 0.45 A	5.43 ± 0.43 A
	C/N	88.07 ± 5.29 A	75.22 ± 8.46 A	83.95 ± 6.85 A	80.22 ± 6.42 A
Root	C	430.32 ± 8.85 AB	442.07 ± 1.21 A	438.03 ± 5.03 AB	422.12 ± 5.70 B
	N	4.34 ± 0.23 C	4.80 ± 0.28 BC	5.61 ± 0.27 B	6.90 ± 0.77 A
	C/N	100.19 ± 4.93 A	93.23 ± 4.81 A	78.71 ± 4.57 B	63.35 ± 6.90 C
Litter	C	430.95 ± 4.41 A	428.60 ± 3.89 A	440.31 ± 4.25 A	451.64 ± 7.23 A
	N	5.43 ± 0.25 B	5.66 ± 0.42 B	7.09 ± 0.48 A	7.20 ± 0.43 A
	C/N	79.92 ± 3.00 A	77.58 ± 6.03 A	62.78 ± 3.34 B	61.77 ± 3.23 B

Treatments: control (CT), drought (D), warming (W), and warming plus drought (WD). The bars with different letters were significantly different from each other (p< 0.05).

### NSC concentrations, iWUE, and C and N stoichiometric characteristics of the role in three parts

**Figure 6:**
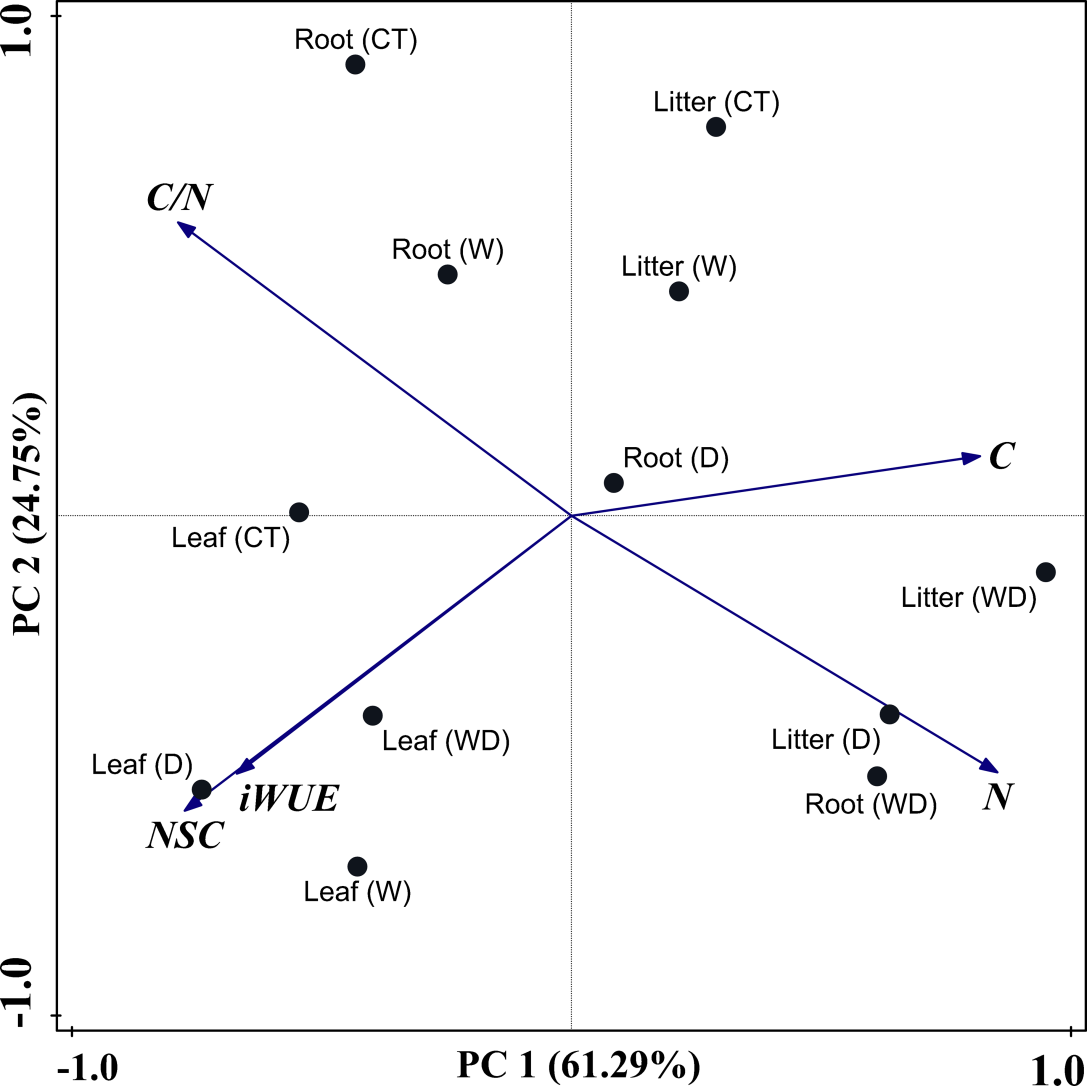
Principal component analysis (PCA) of NSC concentrations, iWUE, C, N concentrations, and C/N ratios in three parts of plant under four treatments.

PCA revealed all the experimental metrics of this study in the four treatments. In our study, the NSC concentrations, iWUE, C and N concentrations, and C/N ratio were used as variables in the different treatments. PC 1 separated the three parts perfectly, showing that leaves were on the left, roots were in the middle, and litter were on the right, which explained 61.29% of the overall variance (**Figure 6**). Cumulative contribution rates of PC 1 and PC 2 were 86.03%.

Among the three parts, the N concentration and the C/N ratio were the most crucial factors in PC 1, where the N concentration and C/N ratio acted in opposite directions and, combined with **Figure 6**, showed that C played a key role. Additionally, NSC concentrations and iWUE were the most important factors in PC 2 (**Table 3**). NSC and iWUE were also indicators of changes in C. Therefore, C was the main physiological factor for adapting to warming and drought environments. The analysis showed that NSC, iWUE, and N elements as well as the C/N ratio could be used as discriminative indicators for *C. lanceolata* in both warming and drought environments, and that the aforementioned indicators jointly regulated C changes to help *C. lanceolata* survive in stressful environments.

**Table 3:** Principal component analysis correlation matrix of physiological indices for three parts of *C. lanceolata* in four treatments.

	PC1	PC2
NSC	−0.142	0.963
iWUE	−0.119	0.837
C	0.502	−0.657
N	0.969	−0.228
C/N	−0.974	0.130

## Discussion

### Effects of warming and drought on NSC and composition

NSC provided substrates for plant growth and metabolism and played a central role in plant responses to the environment (Gersony et al., 2020; Yang et al., 2022). In the present study, the increased NSC in the roots under W treatment was primarily due to the increase in soluble sugar. These results suggested that NSC in the roots is primarily regulated by soluble sugars. Moreover, the study found that warming reduced soluble sugar in the leaves and increased them in the roots, suggesting that soluble sugar in the leaves was transported to the roots under the warming treatment. Because xylem vessels in roots are more susceptible to embolism damage, they need sufficient soluble sugar to participate in xylem embolism refilling to maintain silique swelling pressure (Li et al., 2020). Also, the fact that the ratio of soluble sugar to starch is greater than 1 further confirmed the above conclusion. In addition, drought caused a significant increase in soluble sugar in the litter, suggesting that the leaves occupied a large amount of the growing resource before defoliation. Defoliation can mitigate the negative effects of drought by reducing the consumption of soluble sugars (Quentin et al., 2012; Li et al., 2022). Meanwhile, the increasing NSC in the leaves under W or D treatment was primarily due to the increase in starch that showed that NSC in the leaves is primarily determined by starch in response to warming and drought. However, Adams et al. (2017) and Li et al. (2018) showed that soluble sugar increased while starch decreased in leaves and roots of trees under drought conditions (Adams et al., 2017; Li et al., 2018). Ecologically driven evolution may be generating diversity in starch storage in trees (Furze et al., 2021). Furze et al. (2021) showed that approximately 60% of NSC in the trunk of tree species with more leaves was stored in the form of starch, while only about 35% of NSC is stored as starch in other tree species (Furze et al., 2021). Starch acts as a long-term storage molecule and contributes to the formation of reserves. Stored reserves act as a buffer during warming and drought (Furze et al., 2021). Presumably, NSC is stored mainly in the form of starch in the plant as more leaves fall. The evidence pointed towards stored starch as the main fuel source for survival during and recovery following stressors such as drought and disturbance, and we suggested that the higher starch concentrations associated with deciduousness may confer ability to maintain C balance for the aboveground part of a Chinese fir in a changing environment (Adams et al., 2017; Earles et al., 2018; Smith et al., 2018; Piper and Paula, 2020).

NSC has been shown to be an essential indicator for assessing whether trees are C-starved, yet we have an incomplete picture of how they adapt to changes in NSC storage in a warming and drought environment. In this study, drought caused NSC to rise; however, Signori-Müller et al. (2021) analyzed NSC in leaves and branches of 82 Amazonian tree species across a wide precipitation gradient area and showed a 43% decline in NSC at the most drought-severe sites (Signori-Müller et al., 2021). Yang et al. (2019) found that under conditions of limited C sources (from photosynthesis), plants can and do increase their NSC stores at the expense of normal growth (Yang et al., 2019); the latter research had the same results as ours. Because plant growth would be inhibited before photosynthesis in the face of environmental stress, it leads to the increase of NSC accumulation and storage (Hartmann et al., 2018). Stored NSC provides resilience when the tree’s ability to produce new NSC is impaired by abiotic and biotic stress (McDowell et al., 2008; Carbone et al., 2013; Piper and Paula, 2020). NSC storage by trees at the expense of additional functions, such as growth, is a conservative strategy that may ensure the survival of trees (Sala et al., 2012; Wiley and Helliker, 2012). Therefore, we suggest that when the Chinese fir faces W or D alone, it stores NSC to maintain the C balance at the expense of slower growth. However, Li et al. (2021) studied the interaction of ozone pollution, nitrogen deposition, and drought stress on NSC in poplar leaves and fine roots in temperate climate, and found that plants’ strategies to resist environmental stress were to convert starch in leaves into soluble sugars and transfer NSC in leaves to roots for storage (Li et al., 2021). The strategy is different from ours, considering that this study mainly involved temperature and water, which are different from the stress factors such as those from Li et al. (2021); differences in tree species and climate would also lead to different results.

In addition, this study also found that this strategy failed when combined with warming and drought; soluble sugars in the leaves are significantly reduced, resulting in a slight reduction in NSC. A previous study has shown that when growth is carbon limited, NSC will be used for metabolism and maintenance of growth, and eventually NSC will be reduced (Li et al., 2022). Adams et al. (2009) reported the same result, i.e., that fragrance depleted C reserves at a faster rate in both heat and drought treatments compared to drought alone (Adams et al., 2009). This finding suggests that the combined effects of warming and drought have caused plant growth to be limited by the C starvation crisis, resulting in greater whole-plant carbon loss and potential starvation.

### Effect of warming and drought on iWUE

iWUE is a critical component of water-carbon coupling and process management in terrestrial ecosystems, and a means for trees to adapt to changing environments (Lu et al., 2018). Drought conditions usually lead to an increase in plant iWUE (Kannenberg et al., 2021; Zhang et al., 2022). However, our results showed that W or D alone and the combination of W and D did not affect iWUE in roots, possibly because roots can be effective in avoiding xylem embolism when there is a high availability of mobile soluble carbon compounds (Mcdowell, 2011; Gruber et al., 2012; Sevanto et al., 2014). However, the iWUE of the leaves increased significantly under drought treatment. Stomatal conductance decreased under drought stress (Breshears et al., 2013), which prevented excessive water loss from leaves and thus improved iWUE indirectly. Zhang et al. (2021) found that perennial plantations could heavily control stomata to reduce water loss to prevent hydraulic failure, resulting in growth loss under extreme drought events (Zhang et al., 2022). Maximizing carbon sequestration while minimizing water loss was highly crucial as it raises water stress, and it boils down to a trade-off between carbon uptake and water losses. Meanwhile, our results showed that soluble sugar concentration and NSC concentration were significantly positively correlated with iWUE, indicating that there is no contradiction between plant C storage and water conservation. It suggested that, at this time, *C. lanceolata* was able to regulate the balance of C and water in the body through a growth reduction strategy. Furthermore, both warming and drought significantly increased litter iWUE, indicating that warming and drought had strong effects on the water balance of the leaves. Warming and drought have exacerbated water stress, and defoliation can reduce the adverse effects of water deficit by reducing transpiration (Zhang et al., 2020). Therefore, we believe that defoliation is also a strategy for plants to reduce water loss. This is also confirmed by PCA, which shows that the leaves were mainly affected by iWUE and NSC of the second principal component in both warming and drought environments. Notably, iWUE of the leaves increased under WD in our results (no significant difference), and combined with the analysis of NSC, we suggest that the inability of *C. lanceolata* to mitigate the growth decline caused by climate warming by increasing iWUE under drought stress may lead to severe defoliation and increased mortality.

### Effect of warming and drought on C and N stoichiometric characteristics

C and N stoichiometry is an important indicator for predicting plant productivity; thus, C sequestration might respond to future climate change scenarios (Yue et al., 2017; Sun et al., 2021). Warming and/or drought can change N plant concentration mainly by changing biomass accumulation and N soil availability, thereby also affecting C/N concentration ratios and N use efficiency (Sardans et al., 2008). In this study, drought treatment significantly affected the C/N ratio by increasing the N concentrations of roots and litter. The combined effects of warming and drought significantly affected the concentrations of C in the roots, leading to a decrease in C concentrations. Simultaneously with the PCA, we found that the roots are mainly affected by the values of N and C/N of the first principal component in both warming and drought environments. Several studies have demonstrated that N concentrations increased in roots under drought, and the most probable explanation is that, on the one hand, drought causes root area, root length, and root ramification, and the number of root tips was reduced to facilitate carbohydrate and nitrogen accumulation (Yildirim et al., 2018). On the other hand, it has been found that drought treatment induces plants to allocate more N to their roots to increase their water uptake capacity, thereby reducing the C/N of the roots (Zou et al., 2022). Warming and drought have reduced C concentrations, possibly due to photosynthetic capacity being limited by reduced soil moisture. N is an important element for photosynthesis, and the significant increase in N in the litter suggests that the leaves are capable of carrying out sufficient photosynthesis in front of the fallen leaves. Several leaves can increase photosynthesis, but they also incur significant respiratory and construction costs (Udayakumar and Sekar, 2021). Consequently, defoliation is beneficial for plant C storage. In summary, when plants are under warming and drought conditions, the belowground parts may accumulate N, reducing growth in order to sequester carbon, promote photosynthesis, and conserve water use. Again, the increase of N in the litter verifies the potential benefit of defoliation.

## Conclusion

When plants are exposed to warming and drought, the elements iWUE, C, N, and NSC work together to regulate C and water balance in the plant to adapt to environmental stresses. As warming or drought causes stomata to close, resulting in a trade-off between carbon and water in the plant, the plant chooses to sacrifice growth rate to maintain a higher level of NSC and less water loss in order to maximize carbon sequestration and water conservation strategies. The leaves store more starch to keep the aboveground parts resilient; the higher iWUE and N concentrations in the litter indicate the potential benefit of a defoliation strategy. The roots mainly preserve water and accumulate more soluble sugars and N to conserve water utilization and thus contribute to carbon sequestration in the underground part. The impact of individual species and forest ecosystems on the response to global changes and C storage is highlighted. However, under drought stress, *C. lanceolata* is unable to mitigate the warming-induced decline in growth and continued depletion of C by increasing iWUE, which may result in severe defoliation and increased mortality. The mechanism of carbon and water balance in plants under environmental stress is a complex subject that needs to be explored in greater depth by researchers.

## Data availability statement

The original contributions presented in the study are included in the article/supplementary material. Further inquiries can be directed to the corresponding author.

## Author contributions

YY constructed sample plots and JW designed the experiments. XF, JW and YQX completed sample collection. XF, BZ, YL, XC and YXX conducted the biochemical analyses in the laboratory. XF analyzed data and wrote the manuscript. JW, JZ and TL revised and improved the manuscript. All authors contributed to the article and approved the submitted version.

## Funding

This research was funded by the National Natural Science Foundation of China (31930071) and the Natural Science Foundation of Fujian Province (2021J01146).

## Acknowledgments

We thank the School of Geographical Sciences, Fujian Normal University and Fujian Sanming Forest Ecosystem National Observation and Research Station for providing us with experimental plots and experimental tools in the field. The authors are grateful to the editor and two reviewers for their valuable comments that greatly improved the manuscript.

## Conflict of interest

The authors declare that the research was conducted in the absence of any commercial or financial relationships that could be construed as a potential conflict of interest.

## Publisher’s note

All claims expressed in this article are solely those of the authors and do not necessarily represent those of their affiliated organizations, or those of the publisher, the editors and the reviewers. Any product that may be evaluated in this article, or claim that may be made by its manufacturer, is not guaranteed or endorsed by the publisher.

## References

[B1] Adams H. D. Guardiola-Claramonte M. Barron-Gafford G. A. Villegas J. C. Breshears D. D. Zou C. B.. (2009). Temperature sensitivity of drought-induced tree mortality portends increased regional die-off under global-change-type drought. Proc. Natl. Acad. Sci. U S A. 106, 7063–7066. doi: 10.1073/pnas.0901438106 19365070 PMC2678423

[B2] Adams H. Zeppel M. Anderegg W. Hartmann H. Landhäusser S. Tissue D.. (2017). A multi-species synthesis of physiological mechanisms in drought-induced tree mortality. Nat. Ecol. Evol. 1, 1285–1291. doi: 10.1038/s41559-017-0248-x 29046541

[B3] Auyeung D. Suseela V. Dukes J. S. (2013). Warming and drought reduce temperature sensitivity of nitrogen transformations. Global Change Biol. 19, 662–676. doi: 10.1111/gcb.12063 23504800

[B4] Bonan G. B. (2008). Forests and climate change: forcings, feedbacks, and the climate benefits of forests. Science 320, 1444–1449. doi: 10.1126/science.1155121 18556546

[B5] Breshears D. D. Adams H. D. Eamus D. McDowell N. G. Law D. J. Will R. E.. (2013). The critical amplifying role of increasing atmospheric moisture demand on tree mortality and associated regional die-off. Front. Plant Sci. 4, 266. doi: 10.3389/fpls.2013.00266 23935600 PMC3731633

[B6] Buysse J. Merckx R. (1993). An improved colorimetric method to quantify sugar content of plant tissue. J. Exp. Bot. 44, 1627–1629. doi: 10.1093/jxb/44.10.1627

[B7] Carbone M. Czimczik C. Keenan T. Murakami P. Pederson N. Schaberg P.. (2013). Age, allocation and availability of nonstructural carbon in mature red maple trees. New Phytol. 200, 1145–1155. doi: 10.1111/nph.12448 24032647

[B8] Churakova O. V. Fonti M. V. Siegwolf R. T. W. Saurer M. Myglan V. S. (2020). Impact of recent climate change on water-use efficiency strategies of *Larix sibirica* in the Altai-sayan mountain range. Forests 11, 1103. doi: 10.3390/f11101103

[B9] D'Andrea E. Scartazza A. Battistelli A. Collalti A. Moscatello S. (2021). Unravelling resilience mechanisms in forests: role of non-structural carbohydrates in responding to extreme weather events. Tree Physiol. 41, 1808–1818. doi: 10.1093/treephys/tpab044 33823054

[B10] Danielle E. M. Frederick C. M. David R. W. Katherine A. M. (2016). Thermotolerance and heat stress responses of Douglas-fir and ponderosa pine seedling populations from contrasting climates. Tree Physiol. 37, 301–315. doi: 10.1093/treephys/tpw117 28008081

[B11] Earles J. Knipfer T. Tixier A. Orozco J. Reyes C. Zwieniecki M.. (2018). *In vivo* quantification of plant starch reserves at micrometer resolution using X-ray microCT imaging and machine learning. New Phytol. 218, 1–10. doi: :10.1111/nph.15068 29516508

[B12] Farquhar G. D. Ehleringer J. R. Hubick K. T. (1989). Carbon isotope discrimination and photosynthesis. Annu. Rev. Plant Physiol. Plant Mol. Biol. 40, 503–537. doi: 10.1146/annurev.pp.40.060189.002443

[B13] Farquhar G. D. O'Leary M. H. O. Berry J. A. (1982a). On the relationship between carbon isotope discrimination and the intercellular carbon dioxide concentration in leaves. Aust. J. Plant Physiol. 9, 121–137. doi: 10.1071/pp9820121

[B14] Farquhar G. D. Sharkey T. D. (1982b). Stomatal conductance and photosynthesis. Annu. Rev. Plant Physiol. 33, 317–345. doi: 10.1146/annurev.pp.33.060182.001533

[B15] Fernández de Simón B. Cadahía E. Aranda I. (2022). Aerial and underground organs display specific metabolic strategies to cope with water stress under rising atmospheric CO_2_ in *Fagus sylvatica* L. Physiol. Plant 174, e13711. doi: 10.1111/ppl.13711 35570621 PMC9321914

[B16] Francisco L. Gerard S. Teresa R. Lucía G. Sandra S. M. Anna S.. (2018). Non-structural carbohydrate dynamics associated with drought-induced die-off in woody species of a shrubland community. Ann. Bot-london. 121, 1383–1396. doi: 10.1093/aob/mcy039 PMC6007552 29893878

[B17] Furze M. Wainwright D. Huggett B. Knipfer T. McElrone A. Brodersen C. (2021). Ecologically driven selection of nonstructural carbohydrate storage in oak trees. New Phytol. 232, 1–12. doi: 10.1111/nph.17605 34235751

[B18] Gersony J. Hochberg U. Rockwell F. Park M. Gauthier P. Holbrook N. (2020). Leaf carbon export and nonstructural carbohydrates in relation to diurnal water dynamics in mature oak trees. Plant Physiol. 183, 1612–1621. doi: 10.1104/pp.20.00426 32471810 PMC7401141

[B19] Granda E. Rossatto D. R. Camarero J. J. Voltas J. Valladares F. (2014). Growth and carbon isotopes of Mediterranean trees reveal contrasting responses to increased carbon dioxide and drought. Oecologia 174, 307–317. doi: 10.1007/s00442-013-2742-4 23928889

[B20] Gruber A. Pirkebner D. Florian C. Oberhuber W. (2012). No evidence for depletion of carbohydrate pools in scots pine (*Pinus sylvestris* L.) under drought stress. Plant Biol. 14, 142–148. doi: 10.1111/j.1438-8677.2011.00467.x 21974742 PMC3427021

[B21] Guo X. Peng C. Li T. Huang J. Song H. Zhu Q.. (2021). The effects of drought and re-watering on non-structural carbohydrates of *Pinus tabulaeformis* seedlings. Biology 10, 281. doi: 10.3390/biology10040281 33808347 PMC8066268

[B22] Hararuk O. Campbell E. M. Antos J. A. Parish R. (2019). Tree rings provide no evidence of a CO_2_ fertilization effect in old-growth subalpine forests of western Canada. Global Change Biol. 25, 1222–1234. doi: 10.1111/gcb.14561 30588740

[B23] Hartmann H. Adams H. D. Hammond W. M. Hoch G. Landhäusser S. M. Wiley E.. (2018). Identifying differences in carbohydrate dynamics of seedlings and mature trees to improve carbon allocation in models for trees and forests. Environ. Exp. Bot. 152, 7–18. doi: 10.1016/j.envexpbot.2018.03.011

[B24] Hartmann H. Bahn M. Carbone M. Richardson A. D. (2020). Plant carbon allocation in a changing world-challenges and progress: introduction to a virtual issue on carbon allocation. New Phytol. 227, 981–988. doi: 10.1111/nph.16757 32662104

[B25] Hartmann H. Trumbore S. (2016). Understanding the roles of nonstructural carbohydrates in forest trees-from what we can measure to what we want to know. New Phytol. 211, 386–403. doi: 10.1111/nph.13955 27061438

[B26] Heilman K. A. Trouet V. M. Belmecheri S. Pederson N. Berke M. A. Mclachlan J. S. (2021). Increased water use efficiency leads to decreased precipitation sensitivity of tree growth, but is offset by high temperatures. Oecologia 197, 1095–1110. doi: 10.1007/s00442-021-04892-0 33743068 PMC8591026

[B27] He W. Liu H. Qi Y. Liu F. Zhu X. (2020). Patterns in nonstructural carbohydrate contents at the tree organ level in response to drought duration. Global Change Biol. 26, 3627–3638. doi: 10.1111/gcb.15078 32162388

[B28] IPCC (2021). Climate change 2021-the physical science basis. Chem. Int. 43, 22–23. doi: 10.1017/9781009157896

[B29] Kannenberg S. Driscoll A. Szejner P. Anderegg W. Ehleringer J. (2021). Rapid increases in shrubland and forest intrinsic water-use efficiency during an ongoing megadrought. Pro. Natl. Acad. Sci. U S A. 118, e2118052118. doi: 10.1073/pnas.2118052118 PMC8719875 34930849

[B30] Kannenberg S. A. Phillips R. P. (2019). Non-structural carbohydrate pools not linked to hydraulic strategies or carbon supply in tree saplings during severe drought and subsequent recovery. Tree Physiol. 40, 259–271. doi: 10.1093/treephys/tpz132 31860721

[B31] Katarína M. Ján M. Aleksi L. Giorgio V. Ostrogović S. Augustynczik A.. (2019). Forest carbon allocation modelling under climate change. Tree Physiol. 39, 1937–1960. doi: 10.1093/treephys/tpz105 31748793 PMC6995853

[B32] Li Y. Y. Liu J. X. Zhou G.Y. Huang W. J. and Duan H. L. (2016). Warming effects on photosynthesis of subtropical tree species: a translocation experiment along an altitudinal gradient. Sci. Rep 6, 24895. doi: 10.1038/srep24895 27102064 PMC4840356

[B33] Li M. Guo X. Liu L. Liu J. Du N. Guo W. (2022). Responses to defoliation of *Robinia pseudoacacia* L. and *Sophora japonica* L. are soil water condition dependent. Ann. For. Sci. 79, 1–15. doi: 10.1186/s13595-022-01136-w

[B34] Li W. Hartmann H. Adams H. Zhang H. Jin C. Zhao C.. (2018). The sweet side of global change-dynamic responses of non-structural carbohydrates to drought, elevated CO_2_, and n fertilization in tree species. Tree Physiol. 38, 1706–1723. doi: 10.1093/treephys/tpy059 29897549

[B35] Lin T. Zheng H. Z. Huang Z. H. Wang J. Zhu J. M. (2018). Non-structural carbohydrate dynamics in leaves and branches of *Pinus massoniana* (Lamb.) following 3-year rainfall exclusion. Forests 9, 315. doi: 10.3390/f9060315

[B36] Liu Y. Erbilgin N. Ratcliffe B. Klutsch J. Wei X. Ullah A.. (2022). Pest defences under weak selection exert a limited influence on the evolution of height growth and drought avoidance in marginal pine populations. Pro. R. Soc. B 289, 20221034. doi: 10.1098/rspb.2022.1034 PMC9449467 36069017

[B37] Li Y. Xu Y. Li Y. Wu T. Zhou G. Liu S.. (2020). Warming effects on morphological and physiological performances of four subtropical montane tree species. Ann. For. Sci. 77, 1–11. doi: 10.1007/s13595-019-0910-3

[B38] Li P. Zhou H. Feng Z. (2021). Ozone pollution, nitrogen addition, and drought stress interact to affect non-structural carbohydrates in the leaves and fine roots of poplar. Huan Jing Ke Xue (in Chinese) 42, 1004–1012. 10.13227/j.hjkx.202007213 10.13227/j.hjkx.202007213 33742897

[B39] Lu W. W. Yu X. X. Jia G. D. Li H. Z. Liu Z. Q. (2018). Responses of intrinsic water-use efficiency and tree growth to climate change in semi-arid areas of north China. Sci. Rep. 8, 308. doi: 10.1038/s41598-017-18694-z 29321679 PMC5762888

[B40] Ma Z. Liu H. Mi Z. Zhang Z. He J. (2017). Climate warming reduces the temporal stability of plant community biomass production. Nat. Commun. 8, 15378. doi: 10.1038/ncomms15378 28488673 PMC5436222

[B41] Mccarroll D. Loader N. J. (2004). Stable isotopes in tree rings. Quat. Sci. Rev. 23, 771–801. doi: 10.1016/j.quascirev.2003.06.017

[B42] Mcdowell N. G. (2011). Mechanisms linking drought, hydraulics, carbon metabolism, and vegetation mortality. Plant Physiol. 155, 1051–1059. doi: 10.1104/pp.110.170704 21239620 PMC3046567

[B43] McDowell N. Pockman W. Allen C. Breshears D. Cobb N. Kolb T.. (2008). Mechanisms of plant survival and mortality during drought: Why do some plants survive while others succumb to drought? New Phytol. 178, 719–739. doi: 10.1111/j.1469-8137.2008.02436.x 18422905

[B44] Michael C. D. Anna S. Mariah S. C. Claudia I. C. Joshua A. M. Andrew D. R.. (2014). Nonstructural carbon in woody plants. Annu. Rev. Plant Biol. 65, 667–687. doi: 10.1146/annurev-arplant-050213-040054 24274032

[B45] Mitchell P. J. O'Grady A. P. Tissue D. T. White D. A. Ottenschlaeger M. L. Pinkard E. A. (2013). Drought response strategies define the relative contributions of hydraulic dysfunction and carbohydrate depletion during tree mortality. New Phytol. 197, 862–872. doi: 10.1111/nph.12064 23228042

[B46] Mystakidis S. Davin E. L. Gruber N. Seneviratne S. I. (2016). Constraining future terrestrial carbon cycle projections using observation-based water and carbon flux estimates. Global Change Biol. 22, 2198–2215. doi: 10.1111/gcb.13217 26732346

[B47] Orians C. M. Rabea S. Dukes J. S. Scott E. R. Caroline M. (2019). Combined impacts of prolonged drought and warming on plant size and foliar chemistry. Ann. Bot-london. 124, 41–52. doi: 10.1093/aob/mcz004 PMC6676383 30698658

[B48] Piper F. Paula S. (2020). The role of nonstructural carbohydrates storage in forest resilience under climate change. Curr. For. Rep. 6, 1–13. doi: 10.1007/s40725-019-00109-z

[B49] Quentin A. G. O'Grady A. P. Beadle C. L. Mohammed C. Pinkard E. A. (2012). Interactive effects of water supply and defoliation on photosynthesis, plant water status and growth of *Eucalyptus globulus* Labill. Tree Physiol. 32, 958–967. doi: 10.1093/treephys/tps066 22874831

[B50] Ren M. Liu Y. Qiang L. Song H. Cai Q. Sun C. (2022). Responses of tree growth and intrinsic water use efficiency to environmental factors in central and northern China in the context of global warming. Forests 13, 1209. doi: 10.3390/f13081209

[B51] Sala A. Woodruff D. Meinzer F. (2012). Carbon dynamics in trees: Feast or famine? Tree Physiol. 32, 764–775. doi: 10.1093/treephys/tpr143 22302370

[B52] Sardans J. Peñuelas J. Estiarte M. Prieto P. (2008). Warming and drought alter c and n concentration, allocation and accumulation in a Mediterranean shrubland. Global Change Biol. 14, 2304–2316. doi: 10.1111/j.1365-2486.2008.01656.x

[B53] Seleiman M. F. Al-Suhaibani N. Ali N. Akmal M. Alotaibi M. Refay Y.. (2021). Drought stress impacts on plants and different approaches to alleviate its adverse effects. Plants 10, 259. doi: 10.3390/plants10020259 33525688 PMC7911879

[B54] Sevanto S. McDowell N. G. Dickman L. T. Pangle R. Pockman W. T. (2014). How do trees die? a test of the hydraulic failure and carbon starvation hypotheses. Plant Cell Environ. 37, 153–161. doi: 10.1111/pce.12141 23730972 PMC4280888

[B55] Shi C. G. Silva L. C. R. Zhang H. X. Zheng Q. Y. Xiao B. X. (2015). Climate warming alters nitrogen dynamics and total non-structural carbohydrate accumulations of perennial herbs of distinctive functional groups during the plant senescence in autumn in an alpine meadow of the Tibetan plateau, China. Agric. For. Meteorol. 200, 21–29. doi: 10.1016/j.agrformet.2014.09.006

[B56] Signori-Müller C. Oliveira R. Barros F. Tavares J. Gilpin M. Diniz F.. (2021). Non-structural carbohydrates mediate seasonal water stress across Amazon forests. Nat. Commun. 12, 2310. doi: 10.1038/s41467-021-22378-8 33875648 PMC8055652

[B57] Smith M. Arndt S. Miller R. Kasel S. Bennett L. (2018). Trees use more non-structural carbohydrate reserves during epicormic than basal resprouting. Tree Physiol. 38, 1–13. doi: 10.1093/treephys/tpy099 30219862

[B58] Sun Y. Wang C. Luo X. Qiu N. Ruan H. (2021). Asymmetric responses of terrestrial C:N:P stoichiometry to precipitation change. Global Ecol. Biogeogr. 30, 1–12. doi: 10.1111/geb.13343

[B59] Tang B. Yin C. Wang Y. Sun Y. Liu Q. (2016). Positive effects of night warming on physiology of coniferous trees in late growing season: leaf and root. Acta Oecol. 73, 21–30. doi: 10.1016/j.actao.2016.02.002

[B60] Trowbridge A. Adams H. Collins A. Dickman L. T. Grossiord C. Hofland M.. (2021). Hotter droughts alter resource allocation to chemical defenses in piñon pine. Oecologia 197, 921–938. doi: 10.1007/s00442-021-05058-8 34657177 PMC8591002

[B61] Udayakumar M. Sekar T. (2021). Leaf traits of trees in tropical dry evergreen forests of peninsular India. Ecologies 2, 268–284. doi: 10.3390/ecologies2030015

[B62] Wiley E. Helliker B. (2012). A re-evaluation of carbon storage in trees lends greater support for carbon limitation to growth. New Phytol. 195, 285–289. doi: 10.1111/j.1469-8137.2012.04180.x 22568553

[B63] Xu Z. Shimizu H. Yagasaki Y. Ito S. Zheng Y. Zhou G. (2013). Interactive effects of elevated CO_2_, drought, and warming on plants. J. Plant Growth Regul. 32, 692–707. doi: 10.1007/s00344-013-9337-5

[B64] Yang X. J. Jiang Y. Xue F. Ding X. Y. Cui M. H. Dong M. Y.. (2022). Soil moisture controls on the dynamics of nonstructural carbohydrate storage in picea meyeri during the growing season. Agric. For. Meteorol. 326, 109162. doi: 10.1016/j.agrformet.2022.109162

[B65] Yang B. Peng C. Harrison S. P. Wei H. Wang H. Zhu Q.. (2018). Allocation mechanisms of non-structural carbohydrates of *Robinia pseudoacacia* l. seedlings in response to drought and waterlogging. Forests 9, 754. doi: 10.3390/f9120754

[B66] Yang B. Peng C. H. Zhu Q. A. Zhou X. L. Liu W. G. Duan M.. (2019). The effects of persistent drought and waterlogging on the dynamics of nonstructural carbohydrates of *Robinia pseudoacacia* l. seedlings in Northwest China. For. Ecosyst. 6, 1–17. doi: 10.1186/s40663-019-0181-3

[B67] Yang T. Yang B. Boucher É. Rossi S. (2021). How did climate and CO_2_ concentration affect intrinsic water-use efficiency and tree growth in a semi-arid region of China? Trees 35, 769–781. doi: 10.1007/s00468-020-02075-7

[B68] Yildirim K. Yagci A. Sucu S. Tunc S. (2018). Responses of grapevine rootstocks to drought through altered root system architecture and root transcriptomic regulations. Plant Physiol. Biochem. 127, 256–268. doi: 10.1016/j.plaphy.2018.03.034 29627732

[B69] Yu L. Dong T. F. Lu Y. B. Song M. Y. Duan B. L. (2016). Ecophysiological responses of *Cunninghamia lanceolata* to nongrowing-season warming, nitrogen deposition, and their combination. Photosynthetica 54, 598–610. doi: 10.1007/s11099-016-0647-2

[B70] Yue K. Fornara D. A. Yang W. Peng Y. Li Z. Wu F.. (2017). Effects of three global change drivers on terrestrial C:N:P stoichiometry: a global synthesis. Global Change Biol. 23, 2450–2463. doi: 10.1111/gcb.13569 27859966

[B71] Zhang Z. Wang G. Wang H. Qi Q. He J. S. (2021). Warming and drought increase but wetness reduces the net sink of CH_4_ in alpine meadow on the Tibetan plateau. Appl. Soil Ecol. 167, 104061. doi: 10.1016/j.apsoil.2021.104061

[B72] Zhang Y. Xu G. Peng S. Bai J. Lu Q. Duan B. (2020). Water relations and non-structural carbohydrate responses to the combined effects of defoliation and progressive drought in a dioecious tree. New Forests 52, 605–619. doi: 10.1007/s11056-020-09811-4

[B73] Zhang X. Yu P. Wang D. Xu Z. (2022). Density-and age-dependent influences of droughts and intrinsic water use efficiency on growth in temperate plantations. Agric. For. Meteorol. 325, 109134. doi: 10.1016/j.agrformet.2022.109134

[B74] Zhang Q. Zhou J. Li X. Yang Z. Zheng Y. Wang J.. (2019). Are the combined effects of warming and drought on foliar C:N:P:K stoichiometry in a subtropical forest greater than their individual effects? For. Ecol. Manage. 448, 256–266. doi: 10.1016/j.foreco.2019.06.021

[B75] Zhao J. Bhandari B. Gaiani C. Prakash S. (2021). Physicochemical and microstructural properties of fermentation-induced almond emulsion-filled gels with varying concentrations of protein, fat and sugar contents. Curr. Res. Food Sci. 23, 577–587. doi: 10.1016/j.crfs.2021.08.007 PMC8405962 34485926

[B76] Zhao J. Hartmann H. Trumbore S. Ziegler W. Zhang Y. (2013). High temperature causes negative whole-plant carbon balance under mild drought. New Phytol. 200, 330–339. doi: 10.1111/nph.12400 23822669

[B77] Zheng Y. Guo L. Hou R. Zhou H. Hao L. Li F.. (2018). Experimental warming enhances the carbon gain but does not affect the yield of maize ( *Zea mays* l.) in the north China plain. Flora 240, 152–163. doi: 10.1016/j.flora.2018.02.001

[B78] Zou J. Wu J. Osborne B. Luo Y. (2022). The response of ecosystem carbon and nitrogen pools to experimental warming in grasslands: a meta-analysis. J. Plant Ecol. 15, 733–742. doi: 10.1093/jpe/rtac020

